# Investigating the effects of cerebellar transcranial direct current stimulation on saccadic adaptation and cortisol response

**DOI:** 10.1186/s40673-020-00124-y

**Published:** 2021-01-04

**Authors:** Delia A. Gheorghe, Muriel T. N. Panouillères, Nicholas D. Walsh

**Affiliations:** 1 School of Psychology, University of East Anglia, Norwich, UK; 2 Department of Psychiatry, University of Oxford, Oxford, UK; 3 Department of Experimental Psychology, University of Oxford, Oxford, UK; 4 CIAMS, Université Paris-Saclay, 91405 Orsay Cedex, France; 5 CIAMS, Université d’Orléans, 45067 Orléans, France

**Keywords:** Cerebellum, Saccadic adaptation, Transcranial direct current stimulation, Cortisol

## Abstract

**Background:**

Transcranial Direct Current Stimulation (tDCS) over the prefrontal cortex has been shown to modulate subjective, neuronal and neuroendocrine responses, particularly in the context of stress processing. However, it is currently unknown whether tDCS stimulation over other brain regions, such as the cerebellum, can similarly affect the stress response. Despite increasing evidence linking the cerebellum to stress-related processing, no studies have investigated the hormonal and behavioural effects of cerebellar tDCS.

**Methods:**

This study tested the hypothesis of a cerebellar tDCS effect on mood, behaviour and cortisol. To do this we employed a single-blind, sham-controlled design to measure performance on a cerebellar-dependent saccadic adaptation task, together with changes in cortisol output and mood, during online anodal and cathodal stimulation. Forty-five participants were included in the analysis. Stimulation groups were matched on demographic variables, potential confounding factors known to affect cortisol levels, mood and a number of personality characteristics.

**Results:**

Results showed that tDCS polarity did not affect cortisol levels or subjective mood, but did affect behaviour. Participants receiving anodal stimulation showed an 8.4% increase in saccadic adaptation, which was significantly larger compared to the cathodal group (1.6%).

**Conclusion:**

The stimulation effect on saccadic adaptation contributes to the current body of literature examining the mechanisms of cerebellar stimulation on associated function. We conclude that further studies are needed to understand whether and how cerebellar tDCS may module stress reactivity under challenge conditions.

**Supplementary Information:**

The online version contains supplementary material available at 10.1186/s40673-020-00124-y.

While traditionally, the cerebellum has been primarily associated with motor function and coordination of movement, the consensus today is that it also supports non-motor processing [57]. The cerebellum has a homogenous cytoarchitecture, reciprocal anatomical connections with the cerebral cortex and contains 80% of the total number of brain neurons [20]. Such remarkable characteristics are thought to support the mechanisms of cerebellar involvement in non-motor computations. However, despite a multitude of studies that implicate parts of cerebellar anatomy in higher-order processing beyond motor control, these mechanisms are not fully understood.

The cerebellum may play a role in stress and emotion related processing. Indeed, it has reciprocal monosynaptic connections to the hypothalamic-pituitary-adrenal (HPA) axis [4, 52] and a high density of glucocorticoid receptors [36, 49]. Moreover, cerebellar abnormalities in structure and function have been reported across multiple psychiatric and stress-related disorders [38, 47, 60]. In addition, exposure to severe or mild chronic stress during development is also associated with changes in the cerebellum [13, 61]. Furthermore, individuals with Cushing’s disease who show abnormally elevated levels of cortisol in the blood, demonstrate reduced cerebellar volumes and broad behavioural impairment on cerebellar-dependent and cerebellar-implicated tasks. Neuropsychological testing in Cushing’s disease patients demonstrates broad deficits across memory, attention, reasoning, language and visuospatial domains (see [31] for a review of this literature). All these domains of functioning have been shown to involve the cerebellum, as well as other brain regions e.g. hippocampus, prefrontal cortex [50]. Experimental investigations in Cushing’s patients using more specific, cerebellar-dependent tasks is at present limited. Trace conditioning, a cerebellar dependent process [10], has been shown to be impaired in only one study with Cushing’s syndrome patients [19]. However, similar conditioning impairments in conditioning results have been found following cortisol manipulations in rats [11]. Finally, numerous imaging studies show cerebellar activations during processing of emotional content [56].

Causal evidence of cerebellar involvement in emotional processing exists from studies using non-invasive brain stimulation via either Transcranial Magnetic Stimulation (TMS) or Transcranial Direct Current Stimulation (tDCS). Specifically, cerebellar tDCS was shown to modulate processing of negative emotions on facial features [15]. Furthermore, cumulative changes in mood have been reported as a result of single and repeated active tDCS in healthy individuals [28]. In addition, TMS over the midline cerebellum determined an increase in negative mood when participants were primed with negative images [54], and it was shown to improve negative and affective symptoms in schizophrenic patients [17].

tDCS employs low-intensity constant current via two polarity-dependent electrodes to produce changes in nerve cell membrane excitability [29, 35, 41]. The anodal electrode facilitates an increase in cortical excitability by inducing depolarization of neurons, while cathodal stimulation determines hyperpolarization, leading to a decrease in excitability [6, 45]. Only a limited number of studies have used either tDCS or TMS to investigate the causal relationship between the neuroendocrine response and cortical functioning. Antal et al. [1] applied tDCS of the right medial prefrontal cortex (PFC), prior to a psychosocial stress induction task. This study found a decrease in salivary cortisol levels after anodal stimulation, and an increase after cathodal tDCS in healthy individuals. It was suggested that current-directed endocrine effects were mediated by the anatomical connections between frontal regions of the brain and the hypothalamus [1]. Such polarity-specific changes in cortisol levels were also reported following stimulation of the dorsolateral PFC when participants were presented with negatively-valenced images [8]. Carnevali et al. [9] compared anodal or sham tDCS applied to the left dorsolateral PFC, on autonomic and endocrine responses to a psychosocial stress induction task. In this study, although there were subjective reductions in state anxiety following anodal tDCS there was no effect on stress-induced cortisol release. Recently, Pulopulos et al. [44] used prefrontal high-frequency repetitive TMS (HF-rTMS) to modulate heart-rate and cortisol output to a psychosocial stress task. Results showed that participants in the active HF-rTMS group showed a lower cortisol response to stress. Thus, all prior studies to date have looked at the downregulating effects of the PFC over the activity of the HPA axis. To our knowledge, there are no studies measuring endocrine changes alongside behavioural performance following cerebellar tDCS or TMS. Given the growing evidence for the role of the cerebellum in emotional processing and the stress response, we sought to investigate whether active tDCS stimulation would determine changes in cortisol output.

Cerebellar functioning was evaluated here using a saccadic adaptation task. Adaptation of saccadic eye movements is known to be dependent on the functional integrity of the posterior cerebellum [32]. We have previously shown that the rate of saccadic adaptation is reduced in participants showing high cortisol output following a psychosocial stress-challenge [18]. From this behavioural experiment we were unable to infer the precise underlying neurobiological mechanisms. We speculated that this effect was due to either a direct effect of cortisol that reduced the plasticity of cerebellar neurons, or the effect was due to an indirect effect from anatomically connected regions. For example, this could be due to a reduced motivational effect on the cerebellum from connected regions such as the ventral striatum, whereby experimental reward manipulations have been shown to enhance saccadic adaptation [25]. It could also be due to an effect from the hypothalamus, an important brain region for the control of cortisol, on the cerebellum, as these regions are also anatomically and functionally connected [24, 58]. Therefore, evidence exists that cortisol affects saccade adaptation, however, at present the specific neurobiological mechanisms for this are uncertain. In the current study, we used the same saccadic adaption task from Gheorghe et al. [18], to evaluate cerebellar function together with cortisol levels, following stimulation. During saccadic adaptation, the cerebellum progressively restores optimal motor function when repeated error signals are encountered, by making parametric adjustments to its own fixation error [21, 37, 42].

Therefore, using a single-blind, sham-controlled between-subjects design, we employed cathodal, anodal and sham (control) tDCS during saccadic adaptation, while measuring participants’ cortisol output. We sought to test whether tDCS of the posterior cerebellum influences salivary cortisol levels, and whether this change can in turn influence cerebellar-dependent saccadic adaptation rates, as previously demonstrated. In the context of the limited existing evidence presented above, we predicted that if anodal stimulation determined a decrease in cortisol, this decrease will also be associated with improved saccadic adaptation (and vice versa for cathodal stimulation). An additive effect on cerebellar function was expected, driven by cerebellar stimulation and endocrine consequences.

## Methods

### Participants

Fifty-three participants were recruited through advertisements on participant databases and the local media. Of these, 7 were subsequently excluded from the dataset due to insufficient task trials (> 20% rejected trials). One participant’s cortisol data was >5SD away from the sample mean on all collection time points, and therefore excluded. In total, data was analysed on 45 participants, who had been randomly allocated to one of the following groups: Sham (16 participants; 10 females), Cathodal (14 participants; 8 females), and Anodal (15 participants; 8 females). Participants were right-handed (Edinburgh Handedness Questionnaire [30]), fluent English speakers, aged 18–32 years. All had normal or corrected-to-normal vision (Table 1).

**Table 1:** Participant Characteristics

	Sham	Cathodal	Anodal
N	16	14	15
Age	21.94 (3.85)	21.64 (3.45)	22.53 (4.55)
Gender (females)	10	8	8
BMI	22.39 (2.42)	22.56 (1.87)	21.72 (2.59)
Time of testing	2:22 pm (1:01)	2:47 pm (0:59)	2:10 pm (0:58)
Hormonal contraception (females)	4	3	5
Menstrual cycle (follicular: luteal)	4: 6	5: 2^a^	5: 2^a^
TMD baseline (POMS)	19.37 (23.22)	18.71 (28.43)	16.87 (13.71)
Stressed – Strained baseline (VAS rank)^b^	19.19	26.21	24.07
Calm – Peaceful baseline (VAS rank)	20.91	22.00	26.17
Tense – Pressured baseline (VAS rank)	22.31	27.29	19.73
Satisfied – Content baseline (VAS rank)	25.25	18.54	24.77
Threatened – Vulnerable baseline (VAS rank)	21.84	25.46	21.93
Nervous – Anxious baseline (VAS rank)	20.53	26.18	22.67
Baseline cortisol^c^	0.33 (0.24)	0.50 (0.23)	0.43 (0.29)
Extraversion (BFI - 44)	27.94 (6.47)	25.64 (6.58)	28.67 (6.85)
Agreeableness (BFI - 44)	37.00 (5.45)	33.50 (6.85)	32.20 (6.29)
Conscientiousness (BFI - 44)	34.25 (6.31)	31.86 (5.17)	31.00 (7.43)
Neuroticism (BFI - 44)	20.62 (6.52)	24.14 (6.04)	21.07 (5.93)
Openness (BFI - 44)*	34.25 (7.58)	40.28 (5.62)	34.47 (5.18)
Self-esteem (Rosenberg)	21.87 (5.00)	20.57 (4.52)	19.80 (4.39)
Optimism (SSREIS)	44.50 (3.88)	43.86 (4.75)	41.27 (4.65)
Appraisal of emotions (SSREIS)*	25.12 (1.96)	23.21 (3.31)	21.67 (3.70)
Utilisation of emotions (SSREIS)	15.06 (1.91)	15.21 (2.52)	15.00 (1.89)
Social skills (SSREIS)*	20.37 (2.42)	19.00 (2.96)	17.73 (3.24)
Maternal care (PBI)	30.31 (6.21)	27.07 (7.61)	27.27 (6.98)
Maternal overprotection (PBI)	11.19 (5.78)	14.86 (8.22)	13.67 (6.53)

Unless otherwise specified, numbers depict group averages followed by SD in brackets. ^a^Cycle phase could not be established for two participant. ^b^VAS data shows mean ranks. ^c^Cortisol data depicts log transformed values. *Groups were significantly different, *p* < .05

Study eligibility was evaluated online, screening participants for factors known to affect cortisol levels and tDCS safety. None of the participants had suffered from neurological or psychiatric conditions and had ever taken psychoactive drugs. Furthermore, none suffered epileptic seizures, recurrent fainting spells, loss of consciousness or chronic migraines. There was also no familial history of epilepsy in all participants. Recent or regular intake of any of the following drugs also excluded participants: steroid-based medications, any prescription medication taken for chronic illness or allergies, recreational drugs, anti-malarial treatment. All reported not having any metal fitted to their bodies, no current pregnancies and no history of skin conditions threatening tDCS safety. Three participants had taken part in a brain stimulation study previously (> 1 month). All reported their Body Mass Index within 18–28.

A secondary screening was done at the beginning of the experiment to document further participant information. Twelve females reported use of hormonal contraception. There were 2 reports of secondary amenorrhea (linked to contraception) and therefore menstrual cycle phase was determined for 24 of the 26 female participants. None of the participants had smoked cigarettes, consumed alcohol or had taken any prescription medication or medication affecting cortisol levels or tDCS safety (e.g. psychoactive tablets or drugs) within the 12 h prior to the study. Seventeen participants consumed caffeine within the 12 h prior. All were rested and none had engaged in any intense physical activity within the hour preceding the study.

The study was approved by the local ethics committee in agreement with international regulations.

### Questionnaires

Eligible participants completed state questionnaires measuring mood at the beginning of the experimental session and immediately after tDCS stimulation. A total mood disturbance (TMD) score was calculated based on the Profile of Mood States (POMS) questionnaire [27]. Visual analogue scales (VAS) were also employed. At the end of the study, participants also completed a series of online trait questionnaires and a survey on tDCS adverse effects. The following trait measures were presented in random order (Table 1): the Big Five Inventory (BFI-44) [23]; the Rosenberg Self-Esteem Scale [48]; the Schutte Self-Report Emotional Intelligence Scale (SSREIS) [51]; the Parental Bonding Instrument (PBI) [34]. The adverse effects questionnaire evaluated the following known side-effects, as previously recommended [7]: headache, neck pain, scalp pain, tingling, itching, burning sensation, skin redness, sleepiness, trouble concentrating, and acute mood change. More information about the state and trait measures can be found elsewhere [18]. The self-reported tDCS adverse effects are summarized in Supplemental materials (Tables S1, S2).

### Cortisol assessment

Cortisol levels were determined from saliva using salivettes (Sarstedt Inc., Quebec City, Canada). For collection, participants used a mouth swab, which absorbed saliva for 1–2 min. Samples were centrifuged at 1000 g for 2 min and the resulting material was stored at − 20 °C. Biochemical analyses were performed externally at the University Hospital of South Manchester. Cortisol was analysed via protein crash, using mass spectroscopy. The inter-assay coefficient of variation was less than 10% at 5 nmol/L and the lower limit of quantification was 0.3 nmol/L. There were no values below this threshold.

### tDCS stimulation

**Fig. 1:**
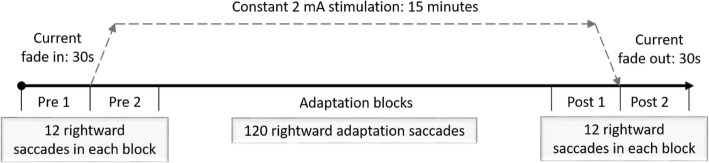
Online stimulation protocol

tDCS was applied using the NeuroConn DC-STIMULATOR PLUS (Rogue Resolutions Ltd., UK). Stimulation was delivered via two rubber electrodes (5x7cm) inserted in saline soaked sponges (approx. 6 mL of solution/side). The active electrode was positioned over the cerebellum, 1 cm below the inion, over the medial line with the lateral edges of the electrode approximately 1 cm away from the mastoid apophysis. The reference electrode was positioned over the right deltoid muscle. Active cathodal or anodal stimulation was delivered online during the saccadic adaptation task at 2 mA for 15 min. The current was gradually ramped up and down over 30s (Fig. 1). The total charge applied during active tDCS was 0.0514 C/cm^2^ and the current density was 0.0571 mA/cm^2^. Sham stimulation was delivered for 30s at 2 mA by placing the anodal electrode over the scalp. In the control (sham) group, the same protocol, including electrode positioning and current ramp times were used, to facilitate effective blinding [29, 59].

### Experimental procedure

**Fig. 2:**
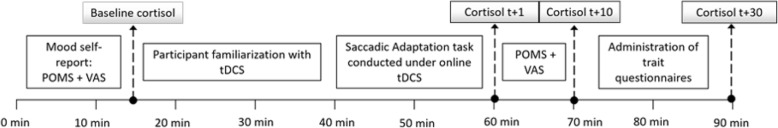
Study protocol

The experimental sessions were single-blind and sham controlled, and they were conducted in the afternoon (1:30 pm–5 pm). Participants’ baseline mood (TMD + VAS) was assessed at the beginning of the session. Approximately 15 min after the start, the first saliva sample was collected (baseline cortisol). Subsequently, the tDCS kit was set up and participants were familiarized with the tDCS procedure and the saccadic adaptation task (1-min practice run). Stimulation was delivered online during the task (Fig. 1). The second saliva sample was collected immediately after the end of the task (cortisol t + 1). In the following 10 min participants completed the mood questionnaires again (POMS+VAS), together with the adverse effects survey. The third saliva sample was collected 10 min after the end of the task (and stimulation) (cortisol t + 10). Following this, participants completed the trait questionnaires. Finally, the fourth saliva sample was collected, 30 min after task end (cortisol t + 30). The protocol is illustrated in Fig. 2. To confirm the effectiveness of the sham, participants’ guesses of their group allocation was tested during the study debrief. None of the participants could determine their study condition. Sham effectiveness was also confirmed by the participants’ reports of tDCS side effects (Supplemental materials).

### Eye tracking protocol and saccadic adaptation

The eye tracking set-up and design of the saccadic adaptation task are detailed elsewhere [18]. Briefly, horizontal movements of the right eye were tracked using the Eyelink 1000 eye tracker (SR Research, Canada). A double-step target paradigm was employed to induce forward saccadic adaptation of rightward saccades via target displacement by 30% of the initial target eccentricity [26]. Leftward saccades were included as distractor trials. In this study, there were 6 sequential blocks: Preadaptation block 1 (24 trials), Preadaptation block 2 (24 trials), two adaptation blocks (2 × 70 trials), Postadaptation block 1 and Postadaptation block 2. Stimulation was turned on just before the start of Pre2 and continued throughout the adaptation sequence and Post1. After this, current was ramped down gradually and Post2 followed without tDCS. The two Pre- and Post-adaptation blocks were employed to evaluate whether stimulation polarity affected baseline metrics and adaptation robustness (via rate of adaptation loss), respectively.

Furthermore, the pre-processing steps employed to treat the eye movement data have also been previously described [18]. We briefly mention that pre-processing was conducted offline using a custom-built Matlab script (Mathworks). Saccades (trials) were inspected manually. There were on average 7.30 ± 5.16% trials per session that were contaminated by artefacts, which were excluded. Saccadic gain, duration, velocity and latency values were computed.

For each variable we also excluded leftward and rightward saccades with values outside +/− 2SDs (mean of 12 trials in either the rightward direction in the pre-, adaptation and post trials, and mean of the 12 trials in the leftward direction in pre-adaptation). There were no group differences in terms of the number of rightward adaptation saccades included in the analysis, following trial rejection and outlier exclusion (F(2,42) = 0.16, *p* > .86). The associated change values in the adaptation and post blocks were calculated relative to preadaptation (e.g.: gain change saccade n = (gain saccade n - mean gain Pre)/mean gain Pre). Subsequently rightward change metrics were averaged in 10 bins of 12 trials, showing change over time. Pre and Post trials were averaged for each saccade direction.

### Statistical analyses

The SPSS Statistics software package was used to perform analyses (IBM, Armonk, NY, USA). The Area Under the Curve with respect to the ground (AUCg) was calculated to yield a measure of total cortisol output. Because most participants showed high cortisol levels at baseline relative to the following collection times, this measure was considered to be most appropriate as its formula is referenced to 0 [43]. Simple group differences on baseline characteristics, trait measures or other relevant variables (e.g., total cortisol output) were evaluated using one-way independent ANOVAs. Kruskall-Wallis tests were employed on ordinal level data or when normality assumptions were violated. Nominal data was evaluated using the Pearson Chi-Square test or Fisher’s Exact Test. Changes over time in saccade metrics or stress variables were investigated using 2 × 2 ANOVAs with Greenhouse-Geisser corrected results where appropriate. Partial eta-squared (η^2^_p_) was reported to describe effect sizes for the repeated-measures ANOVA tests, considering Cohen’s rules of thumb estimating small (.01), medium (.06) and large (.14) effect sizes [12]. To evaluate the steepness of adaptation slopes, a linear slope was fitted to the data over all rightward adaptation trials. Further supplementary results indicating exact statistics are available (Tables S3-S6).

## Results

### Group characteristics at baseline

There were no significant differences between the sham, anodal, cathodal groups on age, Body Mass Index (BMI) and time of testing, F(2,42) < 1.43, *p* > .250. Furthermore, groups were matched on gender (χ^2^(2) = .27, *p* = .874), as well as use of hormonal contraception and cycle phase in the female sample (Fisher’s Exact tests: *p* > .373). Groups did not differ significantly at baseline on (log)cortisol (F(2,42) = 1.68, *p* = .199), TMD (F(2,42) = .05, *p* = .950) and VAS (H(2) < 3.22, *p* > .200). Finally, there were no group differences on most trait measures (F(2,24) < 2.50, *p* > .094). Scores obtained on the openness (BFI-44), appraisal of emotions and social skills (SSREIS) subscales were significantly different between groups (F(2,42) > 3.26; *p* < .048). However, the variance in the total cortisol output (AUCg) could not be significantly explained by group membership (dummy coded factor) (R^2^ = .049, F(2,42) = 1.09, *p* = .347), or by either of the three personality measures when these were additionally entered (together) in the regression model (R^2^ = .123, F(5,39) = 1.09, *p* = .380). We concluded that these baseline differences were unlikely to affect performance on the saccadic adaptation task, as they did not affect participants’ endocrine output. Therefore, differences in adaptation metrics were expected to arise from the experimental manipulation (Table 1).

### Cortisol levels and mood

**Fig. 3:**
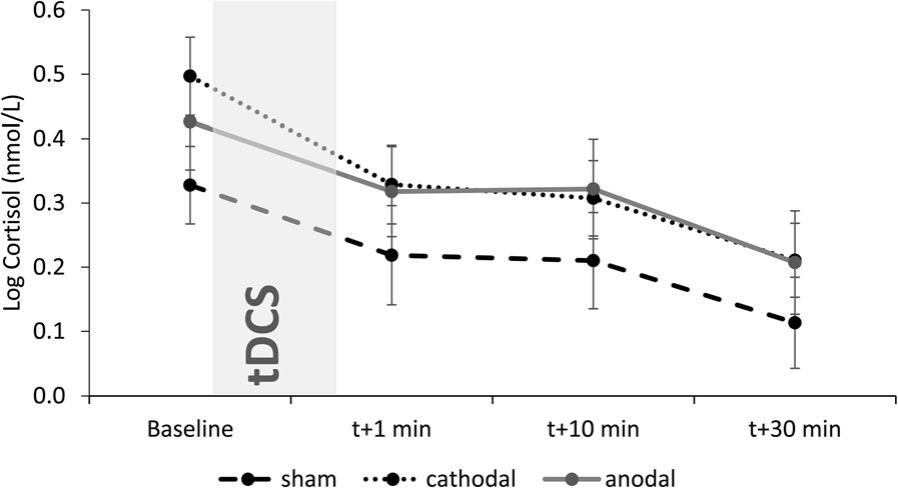
Cortisol change over time. Graph shows log-transformed cortisol levels over four collection time points. No group differences were observed

Logarithmic transformation was applied to normalize the cortisol data (Fig. 3). A 2 × 2 ANOVA with Group factor (Sham, Cathodal, Anodal) and Time (baseline, t + 1, t + 10, t + 30) as the within-subjects factor revealed a main effect of time, F(1,55) = 24.84, *p* < .001, η^2^_p_ = .372. There was no main effect of group (F(1,42) = 1.04, *p* = .361, η^2^_p_ = .047) and no interaction (F(3,55) = .36, *p* = .757, η^2^_p_ = .017). Cortisol levels decreased from the beginning of the experiment to the final sample (t(44) = 6.36, *p* < .001). There were no differences in the total cortisol output (AUCg) amongst the 3 groups, F(2,42) = 1.09, *p* = .347.

We also assessed changes in mood. A 2 × 2 ANOVA with Group factor (Sham, Cathodal, Anodal) and Time (TMD pre-tDCS, TMD post-tDCS) as the within-subjects factor also demonstrated a main effect of time, F(1,42) = 14.69, *p* < .001, η^2^_p_ = .259. There was no group effect (F(1,42) = .07, *p* = .934, η^2^_p_ = .003) and no significant interaction (F(2,42) = 1.77, *p* = .182, η^2^_p_ = .078). Follow-up comparisons showed an overall improvement in mood after tDCS (M = 9.69, SD = 19.30), compared to baseline (M = 18.33, SD = 21.99), t(44) = 3.78, *p* < .001. There were no significant changes in mood on all VAS scales across groups (Wilcoxon ranked tests: Z > -1.34, *p* > .180). Within the cathodal group, participants felt less tense–pressured (M = 1.28, SD = .61) post-tDCS compared to baseline (M = 1.93, SD = 1.07), Z = -2.46, *p* = .014. All other within group comparisons were not significant (*p* > .084).

In summary, tDCS polarity did not affect cortisol levels or subjective mood. There was an overall improvement in mood and a decrease in cortisol output post-tDCS.

### Saccadic baseline performance

We evaluated whether stimulation polarity influenced saccade metrics at baseline. Saccadic gain, duration, velocity and latency were independently submitted to three-way ANOVAs with Block (Pre1, Pre2), Direction (leftward, rightward), as the within-subjects factors, and Group (Sham, Cathodal, Anodal) as the between-subjects factor.

**Fig. 4:**
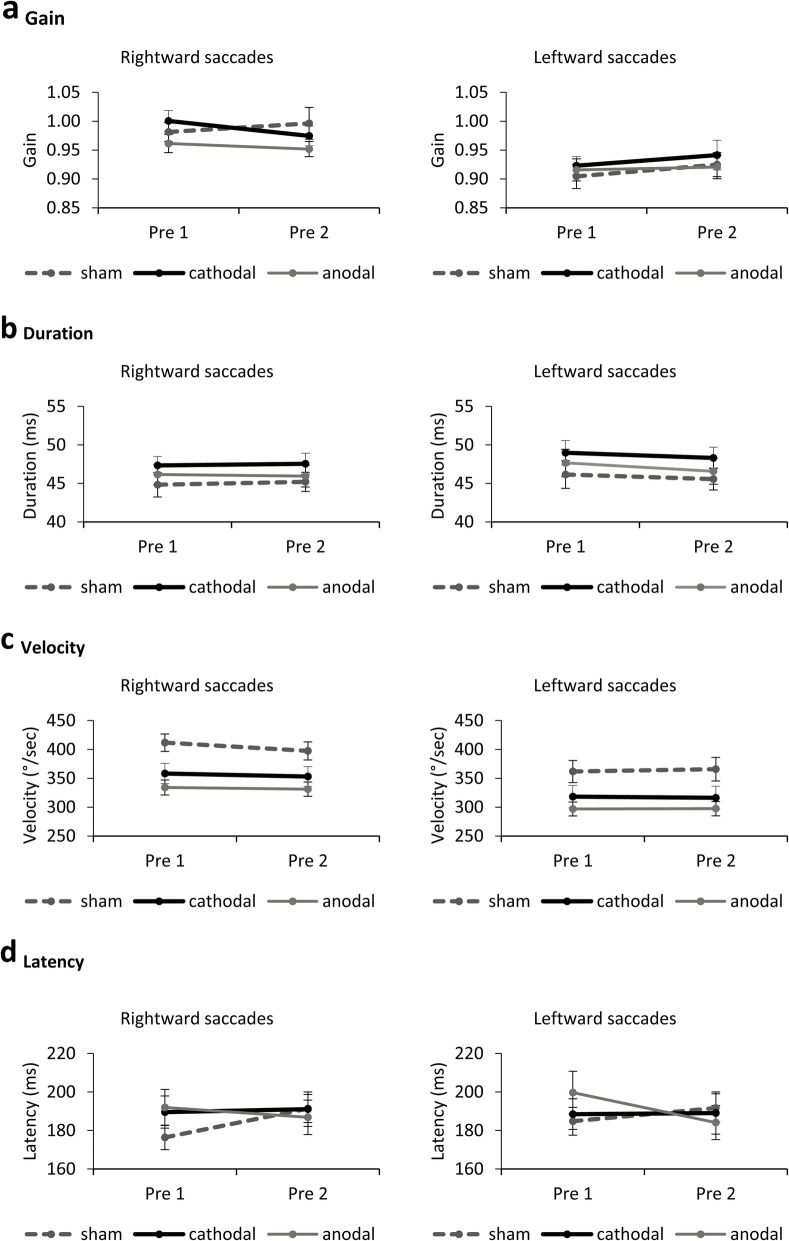
**a**-**d** Baseline performance of saccadic movements. tDCS stimulation polarity did not affect saccadic performance at baseline. Error bars depict SEM

For gain and velocity, analyses revealed main effects of direction (gain: F(1,42) = 17.80, p < .001, η^2^_p_ = .298; velocity: F(1,42) = 62.11, p < .001, η^2^_p_ = .597). Rightward saccades had higher gains across groups and averaged blocks, t(44) = 4.29, p < .001 (Fig. 4a), and higher velocity across averaged blocks in each stimulation group: Sham (t(15) = 4.31, *p* = .001); Cathodal (t(13) = 4.81, *p* < .001); Anodal (t(14) = 4.86, p < .001). Analysis on velocity also yielded a group effect with greater overall velocity in Sham (F(2,42) = 5.31, *p* = .009, η^2^_p_ = .202). Given that the velocity group difference was present when no stimulation was applied (no significant group x block interaction, *p* = .825), we interpret this finding as a pre-existing difference that is independent of stimulation polarity (Fig. 4c). Saccadic duration was also not affected by tDCS polarity and there were no baseline differences (F < 3.19, *p* > .082) (Fig. 4b). Finally, for latency, we found a significant block x group interaction (F(2,42) = 4.95, *p* = .012, η^2^_p_ = .191). However, follow-up comparisons between groups over averaged directions revealed non-significant differences at Pre1 (*p* > .190) or Pre2 (*p* > .545) among the three groups (Fig. 4d).

Given the absence of stimulation polarity effects on baseline adaptation metrics, change values in the adaptation and post-adaptation sequences were calculated relative to the mean preadaptation values (Pre1 and Pre2).

### Effects of tDCS stimulation polarity on adaptation time-course and aftereffects

Adaptation rates were first evaluated by fitting a linear slope to the gain change values of 120 adaptation trials for each participant. No significant differences were found between the adaptation slopes in the sham (M = .05, SD = .08), cathodal (M = .005, SD = .08) and anodal (M = .07, SD = .08) groups (F(2,42) = 2.50, *p* = .094). However, mean values were indicative of milder adaptation slopes in the cathodal group. This was further investigated over 10 time points (bins).

**Fig. 5:**
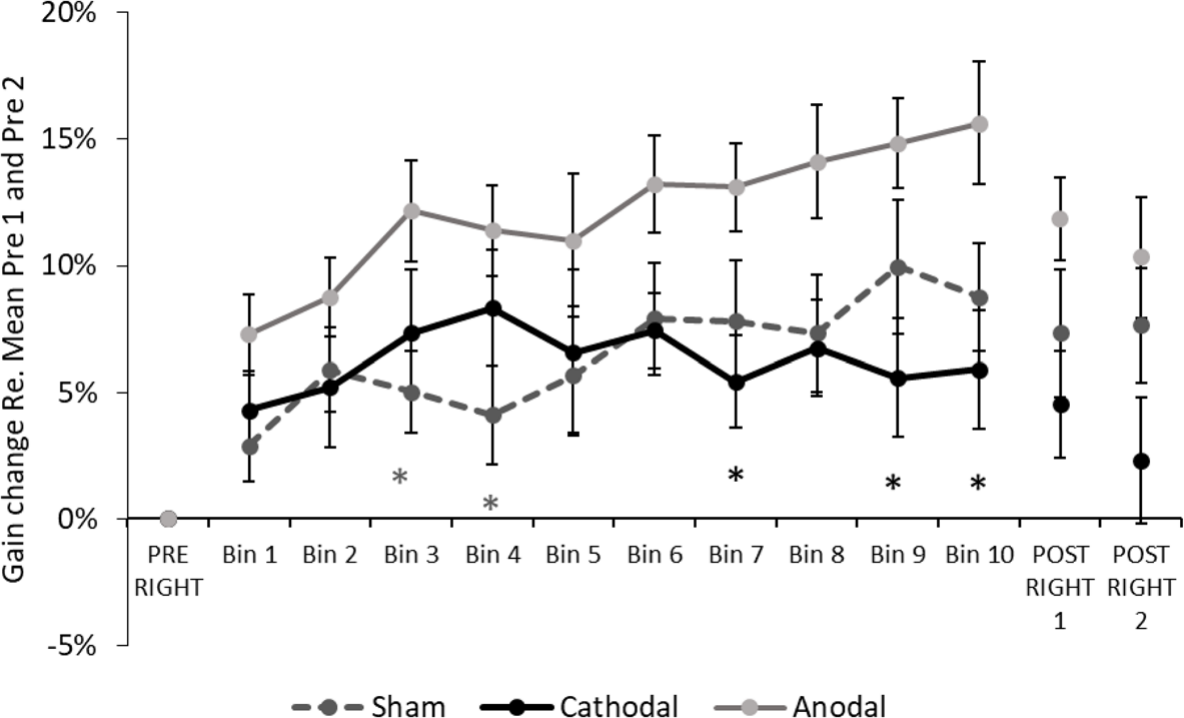
Gain change over time in the three stimulation groups. Significant increase in the Anodal group compared to Cathodal (Bins 7, 9, 10) and Sham (Bins 3, 4); *p < .05. Error bars depict SEM

A two-way ANOVA with Group factor (Sham, Cathodal, Anodal) and Time measured over 10 levels (adaptation bins) showed a progressive increase in saccade size in all groups (time effect: F(4,168) = 5.19, p = .001, η^2^_p_ = .110). Adaptation rates were also significantly different between groups (group effect: F(2,42) = 3.64, *p* = .035, η^2^_p_ = .148). There was no time x group interaction (F(8,168) = 1.52, *p* = .152, η^2^_p_ = .068). Bonferroni-corrected pairwise comparisons were employed to explore group differences throughout the adaptation sequence. Anodal participants had greater gains compared to the Sham group at bins 3 (t(29) = − 2.53, *p* = .046) and 4 (t(29) = − 2.50, *p* = .039). Compared to the Cathodal group, the Anodal group also exhibited higher gain changes at bins: 7 (t(27) = 2.62, *p* = .036); 9 (t(27) = 2.79, *p* = .023); 10 (t(27) = 2.93, *p* = .016). All other comparisons were not significant (*p* > .068) (Fig. 5).

Finally, the postadaptation phase was implemented to evaluate aftereffects in the absence of saccadic error. In a two-way ANOVA evaluating the effects Group (Sham, Cathodal, Anodal) and Time (Post 1, Post 2), gain change was not significantly different between the two blocks (time effect: F(1,42) = 1.12, *p* = .296, η^2^_p_ = .026). Across blocks, we found a significant group effect (F(2,42) = 3.32, p = .046, η^2^_p_ = .137). Group differences were independent of time (interaction: F(2,42) = .50, *p* = .611, η^2^_p_ = .023). As there was no time effect, the two Post blocks were averaged. Bonferroni-corrected comparisons on averaged blocks revealed that aftereffects were significantly greater in the Anodal group compared to the Cathodal group (t(27) = 2.58, *p* = .041). There were no significant differences between the active stimulation groups and participants undergoing sham stimulation (*p* > .517).

To summarize, results suggest that stimulation polarity differentiated between the two active groups in the second half of the adaptation sequence. Compared to the Cathodal group, were changes were small from bin1 to bin10 (1.6%), participants in the Anodal group showed on average an 8.4% increase in gain. This difference was also present in Post. However, increased gain change in the Anodal group was also present early in the adaptation sequence, suggesting higher overall values. Furthermore, saccadic gain under active stimulation was not significantly different from the control group.

## Discussion

The objective of this study was to test whether tDCS over the posterior cerebellum affected cortisol output, mood and cerebellar function in a polarity-dependent manner. Our study built on both the large literature showing that saccadic adaption is a cerebellar-dependent task, as well as our previous study [18] showing that a negative, stressed state reduces saccadic adaptation. We therefore hypothesised that the cerebellum is an important neural locus of this mood effect on saccadic adaptation, and that disruption of the cerebellum via tDCS might disrupt both saccadic adaption, mood and cortisol output. Results showed that anodal stimulation led to an increase in saccadic adaptation, compared to cathodal stimulation. This behavioural effect was driven by the stimulation, as cerebellar tDCS did not influence cortisol levels.

Previous evidence demonstrated that using tDCS to stimulate the prefrontal cortex changes the local excitability of neurons which generates cascading effects on functionally connected areas, such as the hypothalamus, therefore influencing cortisol output [1, 8]. The current study is the first to conduct an evaluation of the endocrine response following direct current stimulation of the cerebellum. Contrary to expectations, we found that polarity-dependent tDCS did not modulate the levels of cortisol or self-reported mood. One likely explanation for this is that tDCS may influence neural activity that is recruited at the time of stimulation [39]. Our study did not involve a stressor or emotionally arousing stimuli, to facilitate task-driven activation of the hypothalamus/HPA axis. It would be this neural activation, which may be susceptible to tDCS (or TMS) modulation. Brunoni and colleagues used emotionally arousing, negative images during online stimulation [8], and both Antal and colleagues, and Pulopulos et al. employed a stress induction task after using either tDCS or TMS to change neural excitability [1, 44]. Whereas similar studies without a stressor do not demonstrate cortisol effects [3]. It is also possible that when applying tDCS (or TMS) on the prefrontal cortex, the electric field is stronger compared to that formed under an electrode placed over the cerebellum. Consequently, the latter configuration requires stronger current intensity to achieve results similar to those observed with cerebral stimulation sites [46].

Saccadic adaptation in the anodal stimulation group achieved greater gain changes compared to the cathodal group. The increase in saccade size was also present in the postadaptation phase, reflecting its robustness. This agrees with the general understanding that by increasing neural excitability during task performance, learning behaviour is facilitated, while decreasing excitability would inhibit behavioural performance [55]. Indeed, online anodal stimulation of the cerebellum was shown to increase the rate of locomotor adaptation, whereas the opposite was found during cathodal tDCS [22]. Cerebellar anodal stimulation also increased the adaptation rate of hand reaching movements relative to sham, anodal occipital stimulation and stimulation of the primary motor cortex [16]. Furthermore, right cerebellar tDCS also determined polarity-specific effects in healthy individuals during acquisition of eye blink conditioning [62].

However, our results are not consistent with all studies on saccadic adaptation. Contrary to the current results, cathodal inhibitory stimulation was previously shown to increase adaptation compared to anodal stimulation, which decreased the rate of learning in healthy individuals [33]. Furthermore, in another study, cerebellar tDCS failed to determine an effect of stimulation on learning [2]. It is possible that these inconsistencies are related to differences in experimental designs [55]. While both studies cited above delivered online stimulation, saccadic adaptation was induced after the machine had been turned on for approximately 11 min [33] and 5 min [2]. Conversely, in the current study, adaptation was elicited approximately 1 min after stimulation began, closer to the beginning of the learning sequence. The issue of timing is of importance considering that it is unclear what the behavioural effects of tDCS are when the stimulated region is not involved in the targeted task [5, 39]. For example, motor learning may be modulated in a polarity-specific manner when stimulation is delivered during the learning sequence, but it may slow down learning regardless of polarity when stimulation is applied before the task [55]. Through “metaplasticity”, the behavioural effects of tDCS may be dependent on the history of the stimulated area [40]. Furthermore, tDCS effects are sensitive to montage and design [29]. Intensity and stimulation duration, as well as the locations of the active and reference electrodes varied between the current and the above-cited saccadic adaptation studies. The current design followed the most recent guidelines published at the time of study [14].

Looking to the future, as discussed above, while active stimulation did not affect cortisol levels or reported affect, it is likely that adding a stressor to the protocol may lead to positive results, suggestive of cerebellar involvement in emotional regulation. More broadly, further studies are needed to investigate the effectiveness of cerebellar tDCS in the treatment of psychiatric symptomatology and the stress response in general [38, 52, 53]. Given that tDCS is a non-invasive technique, it involves low costs, and ease of use, such studies are entirely feasible.

Overall, the current study showed that tDCS delivered to the posterior cerebellum can affect saccadic adaptation in a polarity-dependent fashion, adding to the current evidence that links the posterior cerebellum to this form of learning [32]. Furthermore, anodal stimulation increased the rate of adaptation, as well as retention, compared to cathodal stimulation which determined slower adaptation rates. Therefore, we conclude that cerebellar tDCS directly affects behaviour, but it does not, in a neutral experimental context, directly affect cortisol release or mood.

## Supplementary Information

**Additional file 1.**

## Data Availability

The datasets analysed during the current study are available from the corresponding author on reasonable request.

## Abbreviations

AUCg: Area under the curve with respect to the ground
BFI-44: Big five inventory
BMI: Body mass index
HF-rTMS: High-frequency repetitive transcranial magnetic stimulation
HPA axis: Hypothalamic-pituitary-adrenal axis
PBI: Parental bonding instrument
PFC: Prefrontal cortex
POMS: Profile of mood states
SSREIS: Schutte self-report emotional intelligence scale
tDCS: Transcranial direct current stimulation
TMD: Total mood disturbance
TMS: Transcranial magnetic stimulation
VAS: Visual analogue scales
