# Supplementary material for: Investigating the effects of cerebellar transcranial direct current stimulation on saccadic adaptation and cortisol response
Source: Cerebellum Ataxias. 2021 Jan 4;8:1. doi: 10.1186/s40673-020-00124-y (PMC7784285; doi:10.1186/s40673-020-00124-y)
Supplement: Supplementary file 1 — Additional file 1. [file 40673_2020_124_MOESM1_ESM.docx]

**Supplementary results**

**Adverse effects following tDCS stimulation**

The tDCS adverse effects are summarized in Table S1. Participants reported on the adverse effects, rated their severity, and whether they believed the effects were related to stimulation.

Across groups, tingling (60%), itching (60%), burning sensation (60%) and skin redness (53.3%) were the most common effects, perceived as a definite consequence of tDCS. Sleepiness and trouble concentrating were reported by 46.7% and 28.9% participants respectively (possibly related o stimulation). There were no differences among the three groups, on reported tingling (χ^2^(2)=3.21, p=.200), itching (χ^2^(2)=.84, p=.658), burning sensation (χ^2^(2)=4.25, p=.119), skin redness (χ^2^(2)=1.72, p=.422), sleepiness, (χ^2^(2)=1.16, p=.561) or trouble concentrating (p=.775). Other incidental findings were reported by fewer participants. Two reported having experienced neck pain, which was perceived to be unrelated or only remotely related to tDCS. Two other participants reported scalp pain (definitely/remotely related to tDCS). Other incidental symptoms were noted by one participant in the Cathodal group (soreness - tDCS related) and one in the Anodal group (tickling - tDCS related). Acute change in mood was reported by 3 participants (possibly associated with tDCS). There were no reports of headaches.

With respect to severity, most side-effects were rated as mild (Table S2). Across groups, and for all effect types, side-effects were evaluated significantly more as mild compared to moderate (Z=-4.19, p< .001), and severe (Z=-5.63, p<.001). In two instances, effects were rated as severe for skin redness and trouble concentrating. The redness symptom gradually faded away toward the end of the session and within the subsequent hour. There were no significant differences among the three groups on severity ratings for tingling, itching, burning sensation, skin redness, sleepiness or trouble concentrating (Kruskal-Wallis tests: H(2)<2.43, p>.34.

To conclude, the most common adverse effects were mild sensory stimulation and skin redness under the electrodes. Stimulation polarity did not affect participants’ perception of adverse effects or severity ratings, indicating effective blinding.

**Table S1**

*Occurrence of Adverse Effects following tDCS*

| Adverse Effects | Total (N=45) (%N) | Sham (N=16) (%N) | tDCS related?  (Median) | | | Cathodal (N=14) (%N) | tDCS related?  (Median) | | | Anodal (N=15) (%N) | | tDCS related?  (Median) | Group difference (p value) |
| --- | --- | --- | --- | --- | --- | --- | --- | --- | --- | --- | --- | --- | --- |
| Headache | 0 (0%) | 0 (0%) | | N/A | 0 (0%) | | | N/A | 0 (0%) | | N/A | | N/A |
| Neck pain | 2 (4.4%) | 1 (6.3%) | | 2 | 0 (0%) | | | N/A | 1 (6.7%) | | 1 | | N/A |
| Scalp pain | 2 (4.4%) | 0 (0%) | | N/A | 1 (7.1%) | | | 5 | 1 (6.7%) | | 2 | | N/A |
| Tingling | 27 (60%) | 12 (75%) | | 5 | 6 (42.9%) | | | 5 | 9 (60%) | | 5 | | p = .200^∆^ |
| Itching | 27 (60%) | 11 (68.8%) | | 5 | 8 (57.1%) | | | 5 | 8 (53.3%) | | 4.5 | | p = .658^∆^ |
| Burning sensation | 17 (37.8%) | 6 (37.5%) | | 5 | 8 (57.1%) | | | 5 | 3 (20%) | | 5 | | p = .119^∆^ |
| Skin redness | 24 (53.3%) | 7 (43.8%) | | 5 | 7 (50%) | | | 5 | 10 (66.7%) | | 5 | | p = .422^∆^ |
| Sleepiness | 21 (46.7%) | 6 (37.5%) | | 3.5 | 8 (57.1%) | | | 2.5 | 7 (46.7%) | | 3 | | p = .561^∆^ |
| Trouble concentrating | 13 (28.9%) | 5 (31.3%) | | 3 | 3 (21.4%) | | | 1 | 5 (33.3%) | | 3 | | p = .775 |
| Acute mood change | 3 (6.7%) | 1 (6.3%) | | 4 | 0 (0%) | | | N/A | 2 (13.3%) | | 2.5 | | N/A |
| Others | 2 (4.4%) | 0 (0%) | | N/A | 1 (7.1%) | | | 5 | 1 (6.7%) | | 4 | | N/A |

*Notes.* ^∆^ Values are based on Pearson Chi-Square Tests; the remaining test results refers to Fisher’s Exact Test where expected frequencies were smaller than 5. The median values are based on ratings 1 through 5, i.e., none, remote, possible, probable or definite relation of symptom occurrence to tDCS stimulation; N/A = computation not applicable

**Table S2**

*Severity Ratings of Adverse Effects*

| Adverse Effects | Sham (N=16) | | | Cathodal (N=14) | | | Anodal (N=15) | | | |
| --- | --- | --- | --- | --- | --- | --- | --- | --- | --- | --- |
|  | Mild  (N) | Moderate  (N) | Severe  (N) | Mild  (N) | Moderate  (N) | Severe  (N) | Mild  (N) | Moderate  (N) | Severe  (N) | |
| Neck pain | 1 |  |  |  |  |  | 1 |  |  |  |
| Scalp pain |  |  |  | 1 |  |  | 1 |  |  |  |
| Tingling | 9 | 3 |  | 4 | 2 |  | 8 | 1 |  |  |
| Itching | 6 | 5 |  | 7 | 1 |  | 6 | 2 |  |  |
| Burning sensation | 4 | 2 |  | 6 | 2 |  | 2 | 1 |  |  |
| Skin redness | 6 | 1 |  | 5 | 1 | 1 | 9 | 1 |  |  |
| Sleepiness | 4 | 2 |  | 6 | 2 |  | 4 | 3 |  |  |
| Trouble concentrating | 5 |  |  | 3 |  |  | 4 |  | 1 |  |
| Acute mood change | 1 |  |  |  |  |  |  | 2 |  |  |
| Others |  |  |  | 1 |  |  | 1 |  |  |  |
| Total number of adverse reports | 36 | 13 |  | 33 | 8 | 1 | 36 | 10 | 1 |  |

*Notes.* Table depicts the number of participants who reported adverse effects and the severity ratings for each variable based on raw data. Empty cells show no occurrence of adverse effects. The total number at the bottom of the table shows that most participants rated stimulation side effects as mild and that severity ratings were similar across groups.

**Table S3**

**Results: Group characteristics at baseline** (exact statistic)

|  | Statistic |
| --- | --- |
| Age | F(2,42)=.189, p=.828 |
| Gender (females) | χ^2^(2)=.270, p=.874 |
| BMI | F(2,42)=.543, p=.585 |
| Time of testing | F(2,42)=1.432, p=.250 |
| Hormonal contraception (females) | Fisher’s Test=1.276, p=.689 |
| Menstrual cycle (follicular: luteal) | Fisher’s Test=2.243, p=.373 |
| TMD baseline (POMS) | F(2,42)=.051, p=.950 |
| Stressed – Strained baseline (VAS rank) | H(2)=3.184, p=.203 |
| Calm – Peaceful baseline (VAS rank) | H(2)=1.534, p=.465 |
| Tense – Pressured baseline (VAS rank) | H(2)=3.220, p=.200 |
| Satisfied – Content baseline (VAS rank) | H(2)=2.672, p=.263 |
| Threatened – Vulnerable baseline (VAS rank) | H(2)=2.407, p=.300 |
| Nervous – Anxious baseline (VAS rank) | H(2)=2.228, p=.328 |
| Baseline cortisol | F(2,42)=1.678, p=.199 |
| Extraversion (BFI - 44) | F(2,42)=.814, p=.450 |
| Agreeableness (BFI - 44) | F(2,42)=2.501, p=.094 |
| Conscientiousness (BFI - 44) | F(2,42)=1.078, p=.349 |
| Neuroticism (BFI - 44) | F(2,42)=1.398, p=.258 |
| Openness (BFI - 44) | F(2,42)=4.323, p=.020 |
| Self-esteem (Rosenberg) | F(2,42)=.788, p=.461 |
| Optimism (SSREIS) | F(2,42)=2.275, p=.115 |
| Appraisal of emotions (SSREIS) | F(2,42)=4.997, p=.011 |
| Utilisation of emotions (SSREIS) | F(2,42)=.039, p=.962 |
| Social skills (SSREIS) | F(2,42)=3.259, p=.048 |
| Maternal care (PBI) | F(2,42)=1.062, p=.355 |
| Maternal overprotection (PBI) | F(2,42)=1.131, p=.332 |

**Table S4**

**Results: VAS scales** – changes in mood over time across groups and within groups (exact statistic)

|  | Statistic across groups | Statistic within anodal group | Statistic within cathodal  group | Statistic within sham group |
| --- | --- | --- | --- | --- |
| Stressed – Strained | Z=-.237, p=.813 | Z=-1.100, p=.271 | Z=-1.027, p=.305 | Z=-.276, p=.783 |
| Calm – Peaceful | Z=-.398, p=.691 | Z=-1.020, p=.308 | Z=-.541, p=.589 | Z=-1.354, p=.176 |
| Tense – Pressured | Z=-1.312, p=.189 | Z=-1.000, p=.317 | Z=-2.460, p=.014 | Z=-.541, p=.589 |
| Satisfied – Content | Z=-1.341, p=.180 | Z=-.877, p=.380 | Z=-.566, p=.572 | Z=-1.256, p=.206 |
| Threatened – Vulnerable | Z=-1.153, p=.249 | Z=-1.725, p=.084 | Z=-1.732, p=.083 | Z=-.816, p=.414 |
| Nervous – Anxious | Z=-.834, p=.404 | Z=-.276, p=.783 | Z=-1.414, p=.157 | Z=0, p=.1 |

**Table S5**

**Results: Saccadic baseline performance** (three-way ANOVAs – *p* statistic)

|  | Main effects (*p* value) | | | Interactions (*p* value) | | |  |
| --- | --- | --- | --- | --- | --- | --- | --- |
|  | Block | Direction | Group | Block * Group | Direction * Group | Block * Direction | Block * Direction * Group |
| Gain | .522 | <.001 | .527 | .256 | .538 | .143 | .525 |
| Velocity | .219 | <.001 | .009 | .825 | .900 | .063 | .267 |
| Duration | .511 | .082 | .380 | .899 | .969 | .235 | .999 |
| Latency | .849 | .623 | .896 | .012 | .794 | .205 | .735 |

**Table S6**

**Results: Effects of tDCS stimulation polarity on adaptation time-course and aftereffects**. Bonferroni-corrected *p* values following multiple comparisons at each bin and postadaptation block

|  | Sham vs Anodal | Sham vs Cathodal | Cathodal vs Anodal |
| --- | --- | --- | --- |
| Bin 1 | .133 | 1 | .538 |
| Bin 2 | .830 | 1 | .581 |
| Bin 3 | .046 | 1 | .317 |
| Bin 4 | .039 | .437 | .894 |
| Bin 5 | .500 | 1 | .788 |
| Bin 6 | .161 | 1 | .129 |
| Bin 7 | .207 | 1 | .036 |
| Bin 8 | .089 | 1 | .068 |
| Bin 9 | .404 | .562 | .023 |
| Bin 10 | .113 | 1 | .016 |
| Post Right 1 | .416 | 1 | .067 |
| Post Right 2 | 1 | .365 | .073 |
| Avg. Post (1+2) | .654 | .517 | .041 |
